# Third degree formic acid chemical burn in the treatment of a hand wart: a case report and review of the literature

**DOI:** 10.1186/2193-1801-3-408

**Published:** 2014-08-05

**Authors:** Nicolas Balagué, Philippe Vostrel, Jean-Yves Beaulieu, Jan van Aaken

**Affiliations:** Service of Orthopedic Surgery, Unit of Hand Surgery University Hospitals of Geneva, Geneva, Switzerland

**Keywords:** Wart, Chemical burn, Formic acid

## Abstract

**Objective:**

Cutaneous warts are very common and a large variety of topical treatments and drugs can be employed to cure these skin injuries that can arise on any part of the body. But are these products really safe?

**Method:**

We performed a case description and PubMed literature review using key words “wart,” “chemical burn,” and “formic acid.” All articles in English and French were selected.

**Results:**

This is the first report of a chemical burn by formic acid in the treatment of warts. Numerous topical treatments for cutaneous warts are available with many new drugs appearing every year. However, only a few treatments have proven their effectiveness, such as salicylic acid or cryotherapy with liquid nitrogen that are commonly used. Moreover, most cutaneous warts will resolve spontaneously without any treatment and several products, including topical acids and cryotherapy devices, presented adverse effects such as chemical burns or frostbites so demonstrating that even frequently used treatments can be harmful.

**Conclusion:**

Topical treatments used for wart removal are not without risk even if some products are sold without prescription. For self-treatment products, we recommend enhanced warning by the pharmacist about the risks involved.

## Case presentation

A 58 year-old man in good health presented with a wart on the volar surface of the long finger of his left hand for eight months. A dermatologist recommended a new ointment containing formic acid (FA), accessible without medical prescription and authorized for use in children. This topical chemical agent was to be applied on the wart with a cotton bud, two to three times during the first week of treatment and then once a week until complete disappearance of the wart.

**Figure 1:**
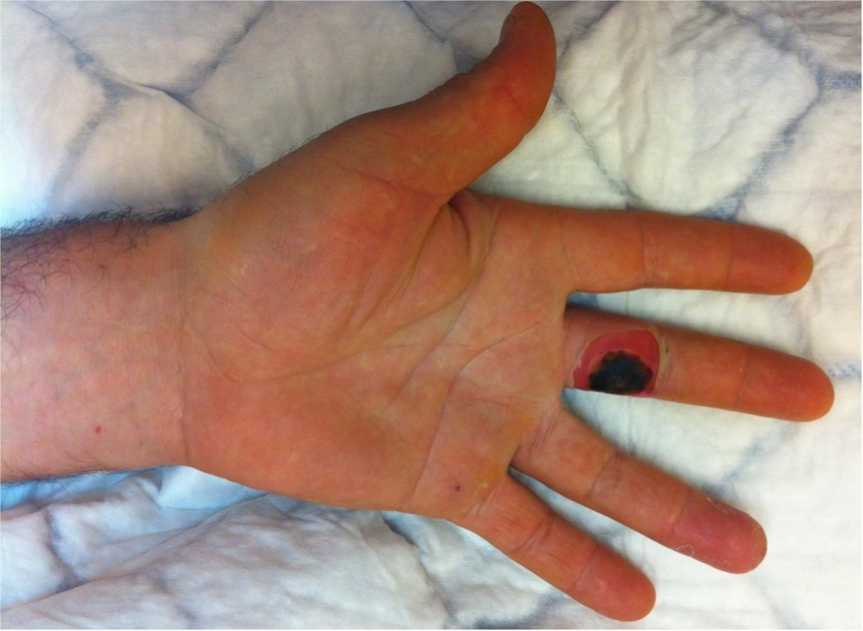
**Initial appearance of the finger with skin necrosis corresponding to a third degree chemical burn.**

Unfortunately the patient did not read the enclosed notice and applied the product to the wart for six continuous hours during the night with use of an occlusive dressing. He was awoken during the night by pain and removed the ointment. In the morning he noted a non-painful skin lesion and two days later presented to the emergency department of our hospital. He had a third degree chemical burn of the ulnar volar side of the first phalanx of the long finger with loss of sensibility on this side of the digit. This lesion presented as a dry skin necrosis of 1.8 cm in diameter with peripheral inflammation without signs of infection (Figure 1). Two point discrimination of the ulnar side of the fingertip couldn’t be perceived (>14 mm).

**Figure 2:**
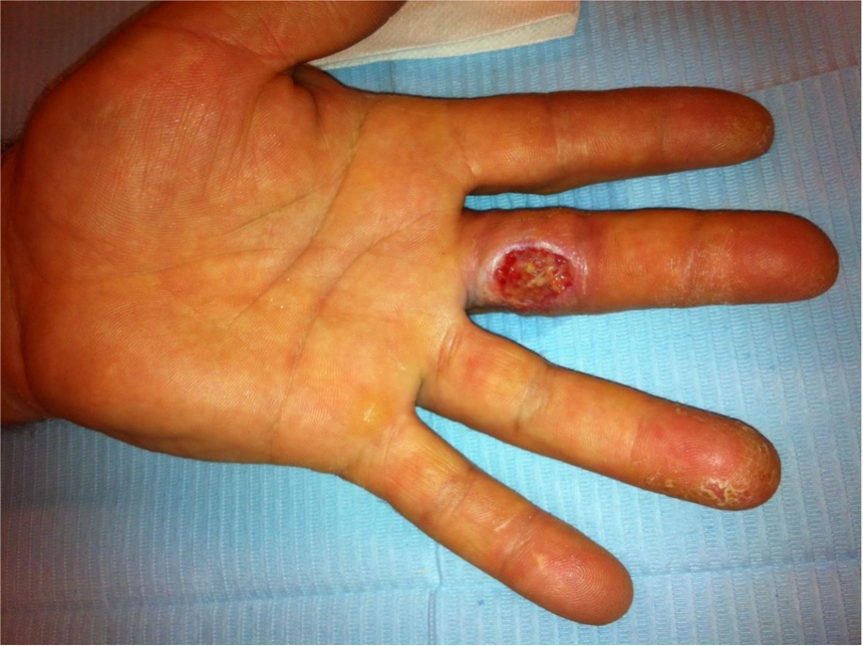
**Appearance of the finger after full thickness skin debridement.**

**Figure 3:**
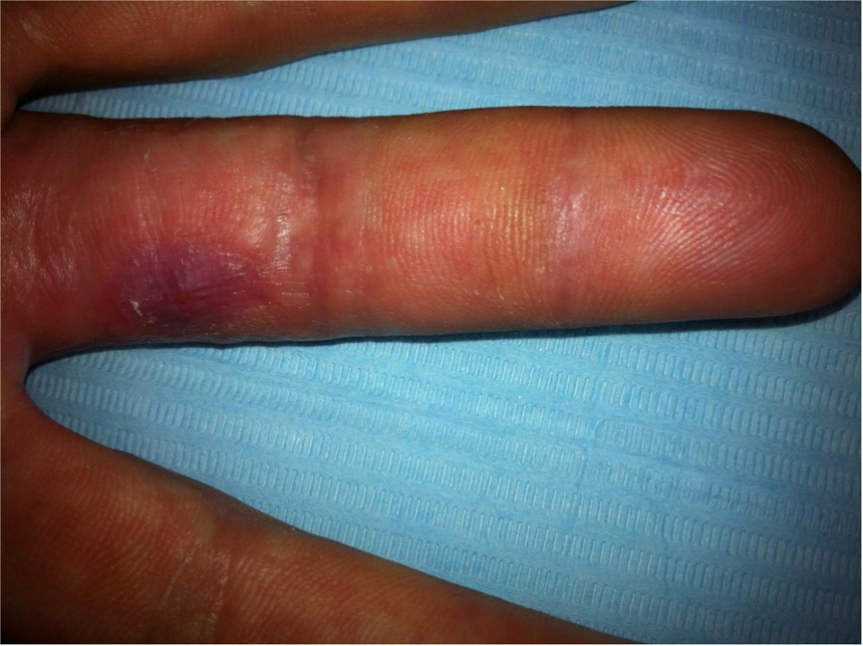
**Appearance of the finger after complete healing.**

**Figure 4:**
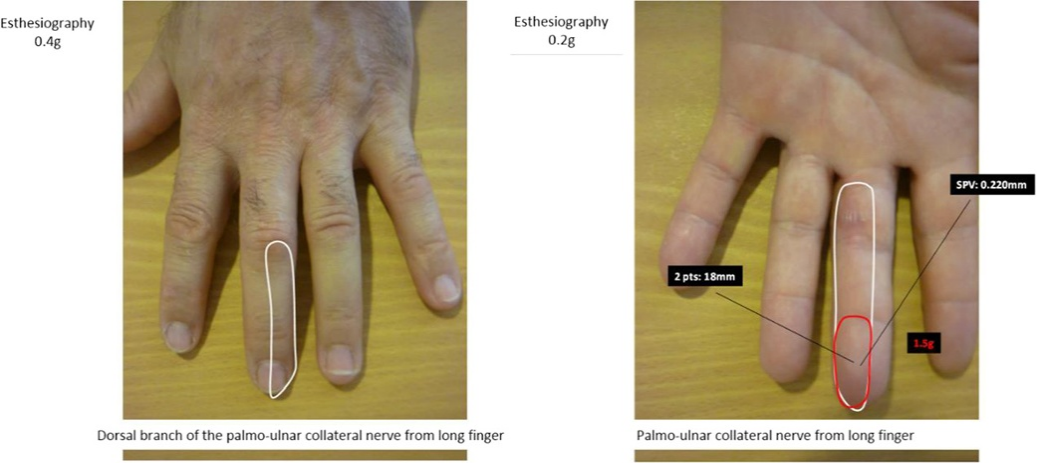
**Sensitivity assessment of the finger at 6 months.**

Under local anesthesia the entire skin thickness along with part of the subcutaneous tissue was excised and the wound left to undergo controlled scaring (Figure 2). During the next weeks the wound became atonic in its center and epithelialization started at the periphery. A flexion contracture of approximately 20° of the proximal interphalangeal (PIP) joint was observed during the scaring process. A second debridement of the subcutaneous tissue was done. The digital canal was intact but the ulnar collateral nerve was found to be exposed. Granulation was then observed during the next few days, and the wound was closed after 58 days of controlled scaring (Figure 3). We decided against skin grafting to improve the aesthetic result and the wound appeared not deep enough to consider a local flap. Complete extension of the PIP joint recovered with use of a 3-point digital splint. Sensibility remained impaired at a 6 month follow-up, with neuropathic pain (tingling, stiffness, numbness) according to the pain questionnaire of Saint-Antoine (French equivalent of the McGill pain questionnaire from Melzack) (Melzack 1975). The assessment of axonal injury was positive: Pressure threshold 1.5 g, 2 point discrimination test 18 mm (normal 2 mm), and vibration threshold 0.220 mm (normal 0.010 mm) (Figure 4).

## Discussion

To the best of our knowledge this is the first report in the literature of a third degree (Devgan et al. 2006) chemical burn of a finger after topical treatment of warts with an ointment containing formic acid.

Warts are caused by some species of human papillomavirus (HPV) and affect adults and children (Lebwohl et al. 2010). They are contagious and usually enter the body through an area of broken skin. They typically occur on the hands and feet, but can appear on any part of the body including face, nails and genitals. Warts often disappear spontaneously after a few months but may last for years and can recur (Micali et al. 2004).

Many products have been tried in the treatment of warts. Often topical application of concentrated salicylic acid or cryotherapy (with liquid nitrogen or carbon dioxide) are used (Bruggink et al. 2010; Bacelieri and Johnson 2005; Gibbs and Harvey 2006; Gibbs et al. 2002; Kwok et al. 2011). Other topical acids include monochloracetic acid, bichloroacetic acid, trichloroacetic acid, lactic acid and formic acid (Gibbs and Harvey 2006; Bhat et al. 2001). Additional therapies include imiquimod, fluorouracil or topical zinc sulfate (Micali et al. 2004; Batista et al. 2010; Khattar et al. 2007). Even systemic therapy has been proposed with the use of cimetidine or levamisole (Simonart and de Maertelaer 2012). Surgical curettage and pulsed-dye or carbon dioxide laser have also be proposed (Park and Choi 2008). However, the majority of these treatments have not definitively proven their effectiveness.

The use of topical acid or cryotherapy for wart removal is not without risk. Cryotherapy devices have been incriminated in frostbite injuries (Ramsey et al. 2007; Sammut et al. 2008), and cases of chemical burn after use of salicylic acid and monochloroacetic acid have been described (Chapman et al. 2006; Tiong and Kelly 2009). Some of the chemical burns were limited to full-thickness skin, but others showed deeper lesions such as tendon rupture (Yates et al. 1988), injuries to the germinal nail matrix, and complete destruction of the growing cartilage (Baser et al. 2008). These cases of deeper injuries were found in children between 10 and 14 years of age.

Formic acid, also called methanoic acid, is found in nature in the venom of ants. It is industrially produced for many uses such as for food preservation since it is antibacterial, for textile production, in some cleaning products, and as a pesticide. In the human body FA is produced from methanol that we ingest, inhale or produce. FA has low toxicity (it is used as a food additive), but when concentrated it is corrosive to biological tissue. This property explains how it is used in the treatment of warts, but can lead to a chemical burn. Direct eye contact with liquid or vapor containing FA can also lead to blindness. It also has a systemic toxicity that can cause acidosis and lead to optic nerve damage and blindness. Furthermore, FA can lead to memory loss, confusion, seizure, coma and cardiac arrest. Chronic exposure may cause kidney or liver damage and skin allergy (Liesivuori and Savolainen 1991).

This case demonstrates that the ability to obtain drugs without medical prescription can cause harm by inappropriate use. In this case the topical application was strictly limited to the surface of the wart, but the duration of the application was not respected since our patient applied the product for 6 hours continuously. A full thickness skin lesion with persistent damage to the underlying collateral ulnar digital nerve resulted, with long lasting loss of ulnar side sensibility of the digit.

## Consent

Informed consent was obtained from the patient for the publication of this report and any accompanying images.

## Abbreviations

FA: Formic acid.
